# Experience of a COVID-19 outbreak response in a general hospital in Gyeonggi Province, Korea

**DOI:** 10.4178/epih.e2021083

**Published:** 2021-10-18

**Authors:** Chanhee Kim, Gawon Choi, Shin Young Park, Jieun Kim, Young Joon Park, Kyungnam Kim

**Affiliations:** 1 Infectious Disease Control Center, Gyeonggi Provincial Government, Suwon, Korea; 2 Korea Disease Control and Prevention Agency, Cheongju, Korea

**Keywords:** Coronavirus disease, General hospital, Disease outbreaks, Korea

## Abstract

**OBJECTIVES:**

Coronavirus disease 2019 (COVID-19) outbreaks in general hospitals are particularly risky because they not only overburden the regional healthcare delivery system, but also increase the possibility of community transmission. This study shares an experience of a COVID-19 outbreak response in a general hospital in Gyeonggi Province, Korea.

**METHODS:**

Since the first COVID-19 confirmed case was recognized in Hospital A on March 29, 2020, the Immediate Response Team of Gyeonggi Province and Korea Disease Control and Prevention Agency devised a plan to conduct an epidemiological investigation and minimize the paralysis of hospital functions. Apart from the epidemiological investigation, a risk assessment of the hospital and management of contacts, including patients and workers, were also undertaken.

**RESULTS:**

In total, 72 confirmed cases were identified, including 26 hospitalized patients, 16 healthcare personnel, 7 visitors, and 22 cases identified externally. The majority of the confirmed cases were exposed in Ward B or were contacts of people exposed in Ward A (58.3% of 72 cases). Among healthcare personnel, caregivers were found to be the most vulnerable to COVID-19 in this outbreak.

**CONCLUSIONS:**

Preparation for all possible situations in medical facilities is important because it is difficult to find alternative resources. The findings of this study provide information on controlling the further transmission of COVID-19 and furnish evidence of the importance of ordinary management skills to be prepared for COVID-19.

## INTRODUCTION

Since the first case of coronavirus disease 2019 (COVID-19) was reported in China on December 31, 2019, a sharp increase in the number of confirmed cases was observed worldwide [1-3]. The first COVID-19 case in Korea, which was confirmed on January 20, 2020, was imported from Wuhan, China [2]. During the COVID-19 pandemic, Korea has experienced several COVID-19 outbreaks that involved hospitals [4-7]. Clusters related to general hospitals are riskier than other clusters because these outbreaks not only overburden the regional healthcare delivery system, but also increase the possibility of community transmission [6,8,9].

The first case of COVID-19 was identified in a general hospital, Hospital A, in Uijeongbu, Gyeonggi Province, on March 29, 2020. Immediately after the detection of the first case, the COVID-19 Immediate Response Team (IRT) of Gyeonggi Province and the Korea Disease Control and Prevention Agency (KDCA) conducted a risk assessment of the case and concluded that it had the possibility to grow into a sizable cluster because most of the contacts were related to Hospital A and nursing homes. This study analyzed the epidemiological characteristics of this hospital-involved COVID19 outbreak and described the efforts of the IRT and KDCA to prevent further transmission of COVID-19.

## MATERIALS AND METHODS

### Recognition of the outbreak

On March 16, 2020, a 75-year-old man with a medical history of hypertension and diabetes mellitus was transferred from a nursing home to Hospital A due to generalized weakness, dysarthria, and dysuria. A computed tomography scan revealed suspicious findings of pneumonia, and the C-reactive protein level was elevated (25.81 mg/L). After medical evaluation of the patient’s neurological symptoms and pneumonia, the patient was admitted to Ward B. During this admission, he underwent 2 COVID-19 polymerase chain reaction (PCR) tests on March 16 and 18, 2020, according to the COVID-19 management guidelines for transferred patients in this hospital, both of which were negative [10]. As the patient’s acute symptoms subsided, he was discharged on March 25, 2020, and was transferred to the nursing home where he had previously been admitted.

After 3 days, on March 29, 2020, he presented to the emergency room of the hospital again with hypotension (53/26 mmHg), dyspnea (oxygen saturation 70% with O_2_ administered at 15 L/min via a simple mask), and fever (37.8°C) aggravated by aspiration pneumonia. As before, a COVID-19 PCR test was performed while the patient was in the emergency room. Even with ventilator care and the use of inotropic drugs, the patient’s septic condition progressed. As his family had signed a do-not-resuscitate form, the patient died on March 30, 2020.

However, the PCR test administered immediately before death was positive. This confirmed case of COVID-19 was immediately reported to the local public health center, and the infection control office started contact tracing and anticipatory screening tests for patients and staff members.

### Management of contacts

According to the COVID-19 management guidelines of the KDCA, contacts should be isolated for 14 days from the day of the last contact with COVID-19 patients [10]. However, for patients and caregivers, the IRT and KDCA decided to define a contact as a person who had visited this hospital since March 24, 2020, the day assumed to correspond to the start of the infectious period (i.e., 2 days before the development of symptoms of the index case, whose initial symptom (myalgia) occurred on March 26, 2020), regardless of whether direct contact with the previously confirmed case was clarified (Table 1). Patients and caregivers were the primary targets of management because of their vulnerability and occupational characteristics. Patients are vulnerable to COVID-19, and caregivers are the closest people to vulnerable patients, even more than patients’ families and nurses. The IRT and KDCA recommended self-isolation for all patients admitted to the hospital. Patients who were admitted to contaminated areas (the 8 wards where the confirmed cases were identified) were strictly recommended to maintain self-isolation for 14 days and were monitored for fever and any other symptoms related to COVID-19 by the public health center of their residence. Among hospitalized patients, those who were classified as contacts were admitted to a previously disinfected ward and placed alone in separate rooms. The healthcare providers who cared for these patients used personal protection equipment during their work. KF94 masks or N95 respirator masks, disposable long-sleeve gowns, gloves, overshoes, and goggles were recommended for moderate-contaminated and low-contaminated areas, whenever they met or contacted these patients.

The exposure classification criteria for doctors, nurses, doctors, other healthcare providers, and other workers differed between patients and caregivers. For nurses and other workers, including nutritionists and cleaners, individuals who worked in moderate-contaminated and high-contaminated areas were classified as contacts, as it was impossible for them to avoid contact with patients (Table 1). Doctors were classified as contacts when there was evidence of them entering a room where a COVID-19 patient was hospitalized. They did so to check their patients who were in the same room as a COVID-19 patient or to provide consultation with other doctors. For other healthcare providers, including pharmacists, radiologists, physical therapists, and sanitary workers, only those who were found to have had direct contact with COVID-19 patients were classified as contacts (Table 1).

The self-quarantine period was decided based on the date of the last visit to the place of contamination, as the contacts were classified according to their place of visit. If the condition of a contact indicated the necessity of hospital treatment, he or she was isolated in a single room of the hospital instead of being discharged home. However, the problem was that it was not possible to disinfect the entire ward while patients were admitted. Hence, the last day of contact (i.e., the day when patients moved from the contaminated area) was repeatedly delayed. Thus, the IRT and infection control team of the hospital established a plan for patient transfer and disinfection of the contaminated area.

Although the COVID-19 KDCA guidelines recommend testing only families of COVID-19 patients and healthcare providers who are identified as contacts, the IRT and KDCA considered that because of their vulnerability, hospitalized patients should also be tested at the end of the isolation period to determine their infection status [10]. The healthcare providers of Hospital A were allowed to resume their work after the 14th day of self-isolation if they had a negative COVID-19 test on the 13th day of self-isolation.

### Risk assessment

In its eighth revision, the KDCA detailed the actions that should be undertaken during COVID-19 outbreaks in medical facilities through its COVID-19 management guidelines [10]. When a COVID-19 outbreak occurs in a medical facility, a risk assessment and management plan should be established based on the COVID-19 management guidelines of the KDCA for the local government (version 8–1) [10]. After identification of the first case in Ward B, the IRT, KDCA, and infection prevention of Hospital A defined Wards A and B as contaminated areas, as there was no physical barrier between the 2 wards. Furthermore, the decision was made to start screening all admitted patients and caregivers for COVID-19, regardless of the ward, considering their vulnerability. Among healthcare providers, only workers in Wards A and B were tested because their scope of movement during work was limited.

On the next day (March 31, 2020), 9 confirmed cases of COVID-19 were reported from other units of the hospital. Therefore, the contaminated area was expanded to Wards C and D. Finally, as more confirmed cases were identified, the risk evaluation of the hospital graded areas as high-contaminated, moderate-contaminated, and low-contaminated (Table 1). The management of patients, caregivers, visitors, and healthcare providers differed according to the grade of contamination.

### Management of the hospital

From the time when the first case of COVID-19 was recognized in this hospital, the common goal of the IRT, KDCA, and hospital was to reopen the hospital by containing the outbreak at the smallest possible scale within the shortest possible period. First, the emergency room and outpatient department were closed to block the influx of new patients. Second, the medical experts at Hospital A separated the department that cared for patients who needed continuous medical treatment. Hence, with the complete closure of the rest of the hospital, only the delivery room (only for emergent patients), endoscopy center (only for emergent patients), radiation treatment center, chemotherapy center, trauma treatment center, and hemodialysis unit were opened carefully. Until the end of the outbreak, the IRT and Hospital A staff closely communicated regarding the step-by-step plan to reopen the hospital.

### Investigation of confirmed cases

Confirmed cases of COVID-19 were defined as patients who had positive results of the COVID-19 PCR test. The IRT and KDCA collected demographic and epidemiological information, including sex, age, medical history, presence of symptoms, onset of symptoms, and other risk factors, through in-depth interviews or reviews of medical records of confirmed COVID-19 cases with the standardized epidemiological investigation form of Gyeonggi Province. If in-depth interviews were impossible because of patients’ medical condition (intubation or death), the IRT interviewed their families or colleagues to collect the information. Closed-circuit television and global positioning systems were important tools in the investigation. The IRT also utilized electronic medical records (EMRs) for contact tracing. Components of the medical records such as progress notes, nursing records, picture archiving and communication system records, order communication system records, and consultation notes were helpful to describe the in-hospital movement history of patients. Moreover, the infection control team provided made this process smoother and more convenient by developing an epidemiological investigation support system in their EMR, which listed the departments that patients visited by date.

Through these tools, the IRT was able to identify an objective basis for making epidemiological decisions. Another challenge during the investigation was posed by asymptomatic confirmed cases. A review of medical records was helpful in investigating these patients. As symptoms are subjective, their intensity differs from person to person, and some individuals are unaware of their symptoms.

### Statistical analysis

With collected data from the results of the epidemiological investigation of confirmed cases, demographic and epidemiological information was combined with the contact management system of KDCA, an integrated management system for diseases and public health. To evaluate and identify the epidemiological risk factors for COVID-19, epidemiological indices were extracted through a descriptive analysis.

### Ethics statement

The study protocol was approved by the Institutional Review Board of Korea National Institute for Bioethics Policy (P01-202107-21-002).

## RESULTS

**Figure 1.**
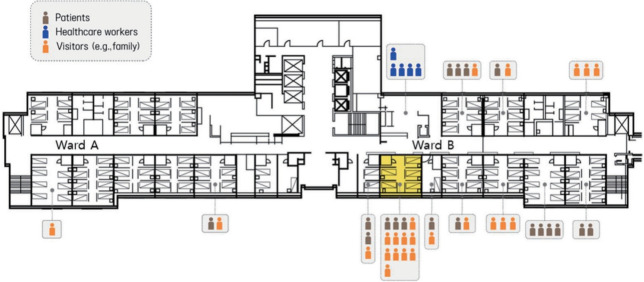
Distribution of coronavirus disease 2019 (COVID-19) confirmed cases in Wards A and B of Hospital A, Gyeonggi Province, Korea, 2020.

Of the 2,562 people in the risk population (976 patients, 627 employees [including healthcare personnel and caregivers], 693 visitors, and 266 others), the IRT identified 72 confirmed cases. Of the 72 confirmed cases, 26 were hospitalized patients, 16 were healthcare personnel, 7 were visitors, and the remaining 23 were identified externally. The suspected source of infection was not established until the outbreak ended. Due to the size of the hospital and its heavy traffic, transmissions between the wards were also not fully elucidated. However, some of the in-hospital transmissions are thought to have occurred by contacts between healthcare providers. Most of the hospital-external cases were infected by household transmission. Monitoring of the outbreak began on the day of the identification of the index case (March 30, 2020) and ended on May 11, 2020, after a process of hospital closure, discovery of additional cases, and disinfection of the hospital (Figure 1).

**Figure 2.**
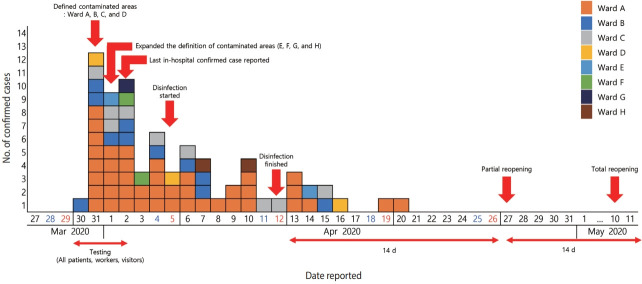
Number of confirmed coronavirus disease 2019 (COVID-19) cases related to Hospital A, Gyeonggi Province, Korea, 2020.

The median age of the confirmed cases was 67 years among patients (range, 9-83), 62 years among healthcare personnel (range, 24-78), 58 years (range, 42-73) among visitors, and 62 years (range, 2-76) among others. Among the 72 confirmed cases, 45 (62.5% of 72) of the confirmed cases were female, although the sex ratio differed from group to group (Table 2). The majority of the confirmed cases were exposed in or were contacts of people exposed in Ward A (58.3% of 72 cases). Most confirmed cases were identified during the first 3 days after the index case was detected by a thorough examination conducted at the hospital (Figure 2). With the exclusion of 3 cases whose symptoms were not available for this report, 50 (72.5% of 69) cases were symptomatic.

The initial symptoms of the confirmed cases are shown in Table 3. Excluding 3 cases whose symptoms were not recorded, 19 cases were asymptomatic and the 50 symptomatic cases had a variety of clinical manifestations. Of the 12 reported symptoms, fever, cough, and sore throat were the most common, reported in 16 (22.2% of 72 cases), 14 (19.4% of 72 cases), and 9 (12.5% of 72 cases) individuals, respectively. Among the remaining symptoms, respiratory symptoms, including sputum production and nasal congestion, were reported in 5 (6.9% of 72) and 2 (2.8% of 72) individuals, respectively. Other accompanying symptoms included chills, myalgia, general weakness, headache, fatigue, dysosmia, dysgeusia, and gastrointestinal symptoms, such as nausea and vomiting.

Although the size of the risk population of caregivers was similar to or larger than that of other occupations, the number of confirmed cases was highest in the caregiver group (Table 4). Furthermore, not only the number of confirmed cases, but also the attack rate was highest among healthcare personnel. Overall, caregivers were revealed to be the most vulnerable group to COVID-19 in this outbreak.

**Figure 3.**
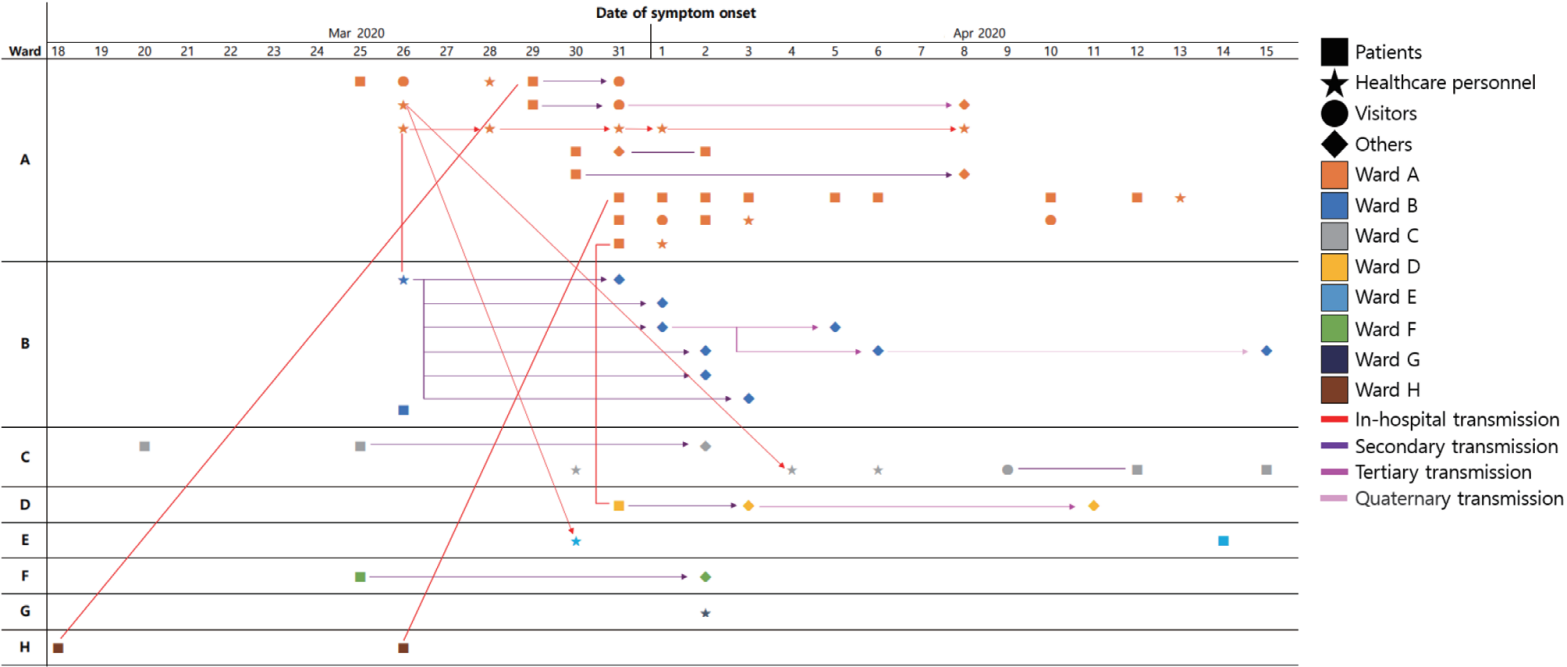
Case map of confirmed cases in Hospital A, Gyeonggi Province, Korea, 2020.

As shown in Figure 3, most of the cases were associated with Wards A and B. However, while most of the cases in Ward A were identified in the hospital, the cases associated with Ward B were confirmed outside of the hospital. Further transmission occurred outside of the hospital mostly among family members; however, successive transmissions related to a caregiver in Ward B occurred in a sauna in Gangwon Province. A caregiver who worked in Ward B from March 18 to March 25, 2020, visited a public bath near her home and spread the virus. Six cases were identified among visitors to the sauna in the public bath, and three more cases were infected by visitors.

The hospital temporarily closed the emergency room and outpatient department after the outbreak started to prevent the influx of new patients. Thus, evacuation of the entire ward was possible, so that all patients who were admitted to the contaminated area were transferred to clean wards. Subsequently, the contaminated area was disinfected. By repeating this process, all patients in the contaminated area were completely moved to a ward where disinfection was completed.

Clean wards were defined as those where no COVID-19 positive cases were reported and had never been visited by COVID-19 positive cases; or those where COVID-19 positive cases had visited or been admitted, but were thoroughly disinfected after transfer of the remaining patients. Patients who could be discharged based on their medical conditions were encouraged to be discharged as soon as possible. For patients who were discharged from a highly contaminated area, their self-quarantine period of 14 days started from the day of discharge.

Disinfection of the contaminated area and transfer of patients occurred between April 4, and April 11, 2020. Therefore, all hospitalized patients began 14 days of self-quarantine by April 11, 2020. Every place where confirmed cases visited, including contaminated areas (wards), intervention rooms, and laboratories, were manually disinfected by fumigation with hydrogen peroxide, and the rest of the hospital was disinfected with 1:100 diluted sodium hypochlorite.

Along with the control measures throughout the hospital, efforts to control and prevent infection in the community were simultaneously made. Among the several groups of the risk population, establishing the whereabouts of caregivers was the most challenging task since there had been no systematic management of caregivers in the hospital. Therefore, the infection prevention team of the hospital worked together with the caregivers’ association to contact discharged caregivers. Moreover, Uijeongbu City Hall sent text messages to the community to guide people who visited Hospital A to actively receive COVID-19 tests. Among the 2,562 people in the risk population, a list of the people who had already left or were discharged from Hospital A was immediately passed to the local health center through the Health and Disease Management System (https://is.kdca.go.kr/) of the KDCA, so that they could receive a COVID-19 test at the nearest local health center to their primary residence.

## DISCUSSION

A general hospital is not only a place where patients are vulnerable to infection [11], but also a place where various types of medical workers who are commute from the community to the hospital move around freely [12,13]. An example of additional transmission related to the in-hospital outbreak is the transmission in this outbreak that occurred in a sauna by a caregiver who worked in Hospital A (Figure 3). The caregiver visited a sauna during the infectious period, which resulted in 8 more confirmed cases. In addition to the risk of community transmission, caregivers also showed a remarkably high attack rate compared to other occupations (Table 4). The caregiver management issue was not only a problem in Korea during the COVID-19 outbreak [14-16], but also during the Middle East respiratory syndrome outbreak in May 2015, in which 40% of confirmed cases were in family members of patients or caregivers [17,18]. Since caregivers are not licensed medical practitioners, they do not have adequate knowledge regarding infection control and personal hygiene practices, and they perform various actions with patients owing to the lack of medical personnel [19]. For example, hospitals prohibited unnecessary conversations between employees or eating together owing to the risk of COVID-19 transmission; however, caregivers often gathered in other areas to meet. Unlike medical practitioners, caregivers do not receive regular education regarding infection control. As we face a new era after the emergence of COVID-19, a wider range of healthcare personnel should be included as targets for infection control programs [14,20].

Furthermore, as most caregivers enter into a contract directly with patients or their families, the role of the hospital is limited to mediation in those relationships. As mentioned before, medical facilities must consider a wider range of workers as targets of management to respond rapidly when COVID-19 confirmed cases are identified [14]. Since most medical facilities in Korea require personal information about their visitors, it will not be difficult to collect information regarding workers in temporary positions or non-regular workers to be prepared for a COVID-19 outbreak. In this outbreak, the difficulty of managing caregivers had a major influence on both the size and the duration of the outbreak.

Another important point of this investigation was risk assessment based on the entire space and health personnel’s occupation. There is no doubt that healthcare providers are at a higher risk of COVID-19 than other workers in the general community [21,22]. Therefore, workers at hospitals should be assessed for risk against more stringent criteria. For example, if the IRT applied the same classification criteria as that of doctors to find the contacts of caregivers, the majority of caregivers, who were later identified as confirmed cases, would have been excluded from the management target because many of them did not share the exact same room with previously confirmed cases. However, a difficult issue in this context is the need to maintain the function of the medical facility as much as possible and to identify all the possible confirmed cases as contacts to prevent further transmission at the same time. Therefore, this example shows that when a COVID-19 outbreak occurs, it is important to make an epidemiological decision with simultaneous consideration of the crowding, vulnerability, and mobility of users.

This study has a few limitations related to the scope of information that could be collected. Since the residence of the confirmed cases was spread across other cities, their information was partially reflected. For example, information regarding the presence or absence of symptoms was not completely collected, and even if it had been collected, the specific symptoms were not known. Another limitation is the overestimation of asymptomatic cases. Our research was based on information from an in-depth epidemiological investigation report, which was written when the confirmed case was recognized, mostly immediately after the test result was reported as positive. Considering that the available E gene Ct value of the asymptomatic confirmed cases varied between 15.36 and 30.52, and that of the RdrP gene varied between 15.58 and 30.95, the disease progression of the confirmed cases might have changed after their transfer to a nationally designated quarantine hospital even though they were initially classified as asymptomatic cases at the time of the epidemiological investigation. However, information after admission to a nationally designated quarantine hospital is not available because medical records are personal information. Therefore, if available, tracking confirmed cases that were initially classified as asymptomatic should be further researched to overcome the limitations of this study.

Currently, because of the COVID-19 pandemic, most medical facilities, including nursing homes in Korea, have prohibited visitors, and Hospital A sent every caregiver home during the outbreak. We believe that these strategies should have been implemented before the COVID-19 pandemic. Criticism has long been directed towards the Korean cultural practice of a caregiver accompanying a sick person to the hospital [17,23]. It is highly welcome that each medical facility has established a thorough visitor management policy in the “new normal” era. In addition, policies for caregivers need different management methods [9]. Various details of the caregivers, such as their basic information, shift schedule, and patient for whom they are responsible, should be systematically managed, and the information should be shared with the hospital. However, the best approach is to avoid using caregivers whenever possible in acute care hospitals. Acute care hospitals are not good places to organize caregivers with formal staff, so creating a caregiver management system can be demanding for them. Therefore, situations where a caregiver takes care of a patient in an acute care hospital should be avoided, and it is better to complete treatment by transferring the patient to a nursing facility after being cared for by the healthcare provider, until acute care is completed.

If a COVID-19 confirmed case is identified in a medical facility, whether he or she is an employee or a patient, it is important to prepare for all possible situations, considering that it is difficult to find an alternative. COVID-19 outbreaks can cause serious situations for both patients and hospitals, since patients cannot find a medical facility to continue their personalized treatment and hospitals experience shortages of human resources to operate the facility. Although the experience of the COVID-19 response at Hospital A is different from current strategies, this report reveals several problems affecting medical facilities and shows that the initial decisions made by the IRT had an important impact on the outcomes of the outbreak. The findings of this study not only provide information on how to control the further transmission of COVID-19, but also furnish evidence of the importance of ordinary management skills to be prepared for COVID-19.

## Figures and Tables

**Table 1.** Classification of contaminated areas and criteria for classifying contacts according to the place of visits and occupation

Level of contamination	No. of primary cases^1^	No. of additional cases^2^	Sum	Criteria for classifying contacts			
				Patients, visitors or families, caregivers	Doctors	Nurses, nursing assistants	Other healthcare providers
High				People who visited the area since Mar 24	People who visited the same room of the COVID-19 patients	People who worked at this area since Mar 24	People came into direct contact with COVID-19 patients
A	31 (4)	11	42				
B	2	9	11				
C	9	0	9				
D	1 (1)	2	3				
Moderate				People who visited the area since Mar 24	People came into direct contact with COVID-19 patients	People came into direct contact with COVID-19 patients	People came into direct contact with COVID-19 patients
E	2 (1)	0	2				
F	1	1	2				
G	1 (1)	0	1				
H	2 (1)	0	2				
Low	0	0	0	People came into direct contact with COVID-19 patients	People came into direct contact with COVID-19 patients	People came into direct contact with COVID-19 patients	People came into direct contact with COVID-19 patients

COVID-19, coronavirus disease 2019.

1 Double counted cases because of their multiple admission history during the infectious period; Three of them were counted as belonging to Ward A, and 1 case was counted as belonging to Ward H considering their date of confirmation of COVID-19.

2 More than secondary transmission.

**Table 2.** Epidemiological characteristics of coronavirus disease 2019 (COVID-19) confirmed cases in Hospital A

Characteristics	Patients (n=26)	Healthcare personnel (n=16)	Visitors (n=7)	Others (n=23)
Age, median (range), yr	67 (9-83)	62 (24-78)	58 (42-73)	62 (2-76)
Sex				
Male	16 (61.5)	1 (6.2)	4 (57.1)	6 (26.1)
Female	10 (38.5)	15 (93.7)	3 (42.9)	17 (73.9)
Related location				
A	16 (61.5)	10 (62.5)	6 (85.7)	10 (43.5)
B	1 (3.8)	1 (6.2)	0 (0.0)	9 (39.1)
C	4 (15.4)	3 (18.7)	1 (14.3)	1 (4.3)
D	1 (3.8)	0 (0.0)	0 (0.0)	2 (8.7)
E	1 (3.8)	1 (6.2)	0 (0.0)	0 (0.0)
F	1 (3.8)	0 (0.0)	0 (0.0)	1 (4.3)
G	0 (0.0)	1 (6.2)	0 (0.0)	0 (0.0)
H	2 (7.7)	0 (0.0)	0 (0.0)	0 (0.0)
Presence of symptoms				
Symptomatic	16 (61.5)	11 (68.7)	7 (100)	16 (69.6)
Asymptomatic	9 (34.6)	5 (31.2)	0 (0.0)	5 (21.7)
Unknown	1 (3.8)	0 (0.0)	0 (0.0)	2 (8.7)

Values are presented as number (%).

**Table 3.** Summary of symptoms of coronavirus disease 2019 (COVID-19) confirmed cases in Hospital A

Symptoms	Confirmed cases, n (%)^1^
Fever	16 (22.2)
Cough	14 (19.4)
Sore throat	9 (12.5)
Chills	7 (9.7)
Myalgia	6 (8.3)
Sputum production	5 (6.9)
General weakness	4 (5.6)
Headache	3 (4.2)
Fatigue	2 (2.8)
Nasal congestion	2 (2.8)
Dysosmia, dysgeusia	2 (2.8)
Nausea, vomiting	1 (1.4)

1 If a single patient showed several types of symptoms, it was counted as duplicate.

**Table 4.** Attack rates among healthcare personnel by occupational group in Hospital A

Variables	Doctor	Nurse	Others	Caregiver
No. of confirmed cases	2	3	2	9
Contacts (size of risk population)	104	193	223	107
Attack rate (%)	1.92 (0.53-6.74)	1.55 (0.50-4.47)	0.90 (0.25-3.21)	8.41 (4.49-15.22)^1^

1 Using the Fisher exact test, the attack rate of caregivers showed statistically significant differences from other occupations.
